# Modelling Ephemeral Gully Erosion from Unpaved Urban Roads: Equifinality and Implications for Scenario Analysis

**DOI:** 10.3390/geosciences8040137

**Published:** 2018

**Authors:** Napoleon Gudino-Elizondo, Trent W. Biggs, Ronald L. Bingner, Yongping Yuan, Eddy J. Langendoen, Kristine T. Taniguchi, Thomas Kretzschmar, Encarnacion V. Taguas, Douglas Liden

**Affiliations:** 1 Departamento de Geologia, Centro de Investigaciôn Cientifica y de Educaciôn Superior de Ensenada (CICESE), 22860 Ensenada, Mexico; tkretzsc@cicese.mx; 2 Department of Geography, San Diego State University, San Diego, CA 92182, USA, tbiggs@mail.sdsu.edu (T.W.B.), ktaniguchi-w@sdsu.edu (K.T.T.); 3 National Sedimentation Laboratory, Agricultural Research Service, USDA, Oxford, MS 38655, USA, Ron.Bingner@ars.usda.gov (R.L.B.), Eddy.Langendoen@ars.usda.gov (E.J.L.); 4 Office of Research and Development, United States Environmental Protection Agency (USEPA), Research Triangle Park, NC 27711, USA, Yuan.Yongping@epa.gov; 5 Department of Rural Engineering, University of Côrdoba, 14071 Cordoba, Spain, ir2tarue@uco.es; 6 San Diego Border Office, United States Environmental Protection Agency (USEPA), San Diego, CA 92101, USA, Liden.Douglas@epa.gov

**Keywords:** gully erosion, unpaved roads, AnnAGNPS model, model equifinality, urbanization, watershed management

## Abstract

Modelling gully erosion in urban areas is challenging due to difficulties with equifinality and parameter identification, which complicates quantification of management impacts on runoff and sediment production. We calibrated a model (AnnAGNPS) of an ephemeral gully network that formed on unpaved roads following a storm event in an urban watershed (0.2 km^2^) in Tijuana, Mexico. Latin hypercube sampling was used to create 500 parameter ensembles. Modelled sediment load was most sensitive to the Soil Conservation Service (SCS) curve number, tillage depth (Td), and critical shear stress (τ_c_). Twenty-one parameter ensembles gave acceptable error (behavioural models), though changes in parameters governing runoff generation (SCS curve number, Manning’s n) were compensated by changes in parameters describing soil properties (T_D_, τ_c_, resulting in uncertainty in the optimal parameter values. The most suitable parameter combinations or “behavioural models” were used to evaluate uncertainty under management scenarios. Paving the roads increased runoff by 146–227%, increased peak discharge by 178–575%, and decreased sediment load by 90–94% depending on the ensemble. The method can be used in other watersheds to simulate runoff and gully erosion, to quantify the uncertainty of model-estimated impacts of management activities on runoff and erosion, and to suggest critical field measurements to reduce uncertainties in complex urban environments.

## 1. Introduction

Both rural and urban development can increase erosion and the delivery of land-based sediment into receiving water bodies, including estuaries, coasts, and inland lakes and reservoirs. Unpaved roads, in particular, represent one of the principal landscape features of rural urbanization in developing countries. Ephemeral gullying, including on unpaved roads, is an important soil erosion process reported in many environments [1]. Road drainage impacts erosive processes, increasing flow peaks and total discharge [2], which is also observed in monsoonal climates [3].

Ephemeral gullies are important components of sediment budgets in both natural and human-disturbed environments. The term ephemeral indicates that they are temporary features, commonly removed by tillage operations [4] or filled by road maintenance in urban environments. Ephemeral gully formation is the product of a complex interaction between terrain topography, climate, soil properties, land cover, and management practices [5], and ephemeral gullies can be the primary source of sediment loss in agricultural and urban environments [6–8].

Semi-arid watersheds are highly sensitive to soil and stream channel erosion following urbanization [9,10]. Gudino-Elizondo et al. [11] observed that gullies in Tijuana, Mexico, formed almost exclusively on unpaved roads, reflecting the role of roads in routing flow and their vulnerability to gully erosion. Unpaved roads can also be an important component of anthropogenic sediment generation in the study area [11], as has also been reported in other settings [12], including logged forests [13,14] and tropical islands [15].

There are few studies assessing gully erosion in urban settings, as documented in a compilation of gully erosion studies by Castillo & Gomez [16]. Adejiji et al. [8] described the relationship between urban surface characteristics and gully erosion in Nigeria, and found a significant relationship between soil texture, land use, and gully erosion. However, measurements and modelling of ephemeral gully erosion rates on unpaved roads have rarely been conducted in urban watersheds. Control of gully erosion could involve road paving, but at the cost of increasing peak discharge. Other best-management practices (BMPs) include revegetation of hillslopes that produce runoff, which could mitigate both runoff and sediment production, but this strategy has not been quantitatively evaluated. The trade-off between sediment control and runoff production is particularly important, but remains unquantified.

Numerical models have been used to simulate soil and gully erosion rates [17–20] and to assess the impacts of conservation practices [21]. These models differ in terms of their structure, their assumptions and the input data necessary for model calibration and application [22]. Bull and Kirkby [23] reviewed the conditions for gully formation and noted that gully modelling must be based on the relationship between flow hydraulics and soil properties [24]. Nachtergaele et al. [25] reported a good performance of the Ephemeral Gully Erosion Model (EGEM) predicting gully volumes in agricultural areas of Spain and Portugal.

The Annualized AGricultural Non-Point Source (AnnAGNPS) model was developed to simulate sheet and rill erosion in agricultural environments [19,20], and has been utilized and validated in many studies, including in evaluations of the impact of agricultural BMPs [26–32]. Gordon et al. [33] improved on the EGEM using more process-based techniques and this revised EGEM has been incorporated in AnnAGNPS [20]. Improvements to the gully widening approach within AnnAGNPS were developed by Bingner et al. [20]. Head-cut migration rates within the model are based on physical approximations of mass, momentum, and energy transfer, described by Alonso et al. [34]. The AnnAGNPS model has not been tested to simulate and monitor ephemeral gully erosion rates in an urban context.

In hydrologic and soil erosion modelling, several parameter sets may adequately simulate the observed behaviour of the system; such models are called “behavioural” [35]. Hornberger and Spears [36] rejected the idea of an optimal model structure or parameter set in favour of multiple parameter combinations, which all provide acceptable fits to observed data, called equifinality by Beven [37]. Equifinality suggests that there are multiple interactions among the parameters within a model to produce simulations that may be equally acceptable. Equifinality is especially important when simulating the impacts of changes in climate, land use, or watershed management, since different parameter ensembles can generate different predictions under change [35]. Field measurements may be taken to constrain model parameters, but those measurements may or may not match the parameters obtained through calibration due to either unsampled heterogeneity, problems with model structure or to other processes operating at spatial scales larger than that of the field measurements. To our knowledge, no study has addressed equifinality in gully erosion modelling and its impact on scenario analysis, particularly in an urban setting.

This paper aims to generate a set of behavioural gully erosion models in a rapidly urbanizing watershed, and to explore the impact of parameter uncertainty on scenario analysis in a practical management context. We address the following research questions: (a) How well does the AnnAGNPS model predict urban gully erosion? (b) What are the most sensitive AnnAGNPS parameters in urban gully erosion modelling? And (c) What are the implications of parameter uncertainty for evaluation of the impact of road paving and other BMPs on runoff and erosion? The study is novel in terms of evaluating AnnAGNPS’s capabilities in assessing gully erosion in urban watersheds, which included using a high-horizontal-resolution (30 cm cell size) Digital Elevation Model (DEM) generated using a combination of Unmanned Aerial Systems (UAS) and Structure from Motion (SfM) photogrammetric techniques [11] to improve representation of topographic attributes and flow routing to predict ephemeral gully formation. Understanding the process of gully erosion will be critical in describing and quantifying sediment production within urbanized watersheds, and consequent loads of water and sediment to ecosystems downstream.

## 2. Materials and Methods

### 2.1. Study Area

**Figure 1.**
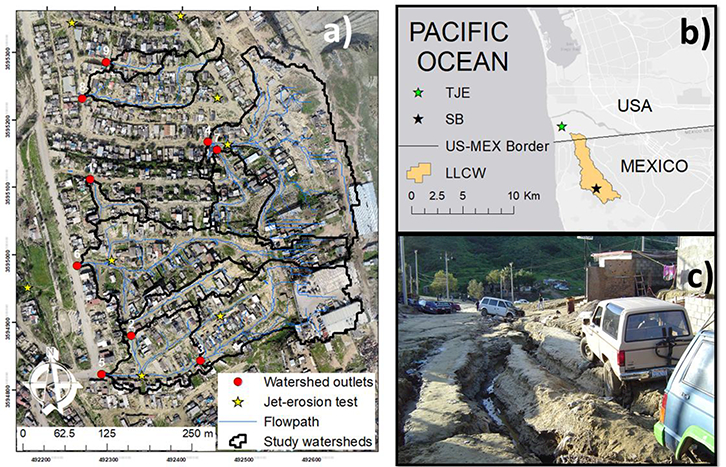
**(a)** UAS-SfM-derived orthophoto for San Bernardo (SB), and the 9 study watersheds with their outlets; **(b)** Geographic location of the Los Laureles Canyon Watershed (LLCW), SB, and the Tijuana River Estuarine Reserve (TJE); **(c)** one example of land degradation caused by gully erosion in Tijuana, Mexico.

The San Bernardo (SB) neighbourhood is located within Los Laureles Canyon Watershed (LLCW), a bi-national watershed that flows from Tijuana, Mexico, into the southwestern arm of the Tijuana River Estuary, Imperial Beach, CA, USA. The LLCW drainage area is 11.58 km^2^, with 10.8 km^2^ in Mexico and 0.75 km^2^ in the United States (Figure 1b). The climate is Mediterranean, with a wet season from November to April and annual precipitation of approximately 240 mm per year. Soils in SB are sandy uplifted marine terraces with steep slopes (mean 15 degrees), resulting in high vulnerability to soil and gully erosion [11]. Based on a soils map of San Diego County and samples of soil texture taken in the watershed, the soils are similar to the Las Flores soil group, which are described as having loamy sand A horizons with greyish brown and light brownish grey colour, and a sandy clay B horizon grading to weakly consolidated siliceous marine sandstone in the C horizon [38]. SB has typical mixed urban-rural land cover (Figure 1a) with high population density (~6500 people-km^−2^). SB was urbanized in 2002, and has unauthorized housing developments (“invasiones”). The construction of unpaved roads on highly erodible soils enhances gully formation, affecting the quality of life for the residents [39], and is likely a significant contributor to total sediment production at the watershed scale. The gully network in SB is filled in with sediment at specified dates to represent road repair. However, road repair was not important, because gully formation was simulated from a single storm event.

Excessive erosion, transport and deposition of sediment have many detrimental effects on the people living in the watershed (Figure 1c) and have impaired conditions for aquatic life in the Tijuana River Estuary (Figure 1b). The Tijuana River National Estuarine Research Reserve, located in the United States, is listed as “impaired” by the State of California due to excessive sediment loads [40]. Several U.S. government agencies spend approximately $3 million per year to remove sediment produced in Mexico [41], and it is therefore important to quantify soil erosion rates in the upper watershed in order to identify cost-effective solutions to reduce sediment loads into the Estuary.

### 2.2. Observed Gully Erosion

**Figure 2.**
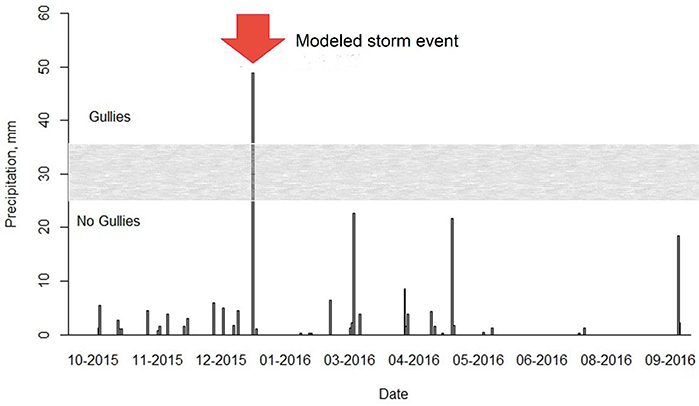
Daily rainfall time series for the 2016 water year. The grey box represents the rainfall threshold (~25–35 mm) for gully formation observed in the study area.

Both ground- and UAS-based surveys of a gully network that formed in SB following a large storm event on 5–7 January 2016 were conducted on 16 January 2016 (Figure 2). The storm was the largest of the water year (—50 mm of total rainfall) and had a 15 min maximum rainfall intensity of 4.8 mm, which has a 1 year recurrence interval [42]. Other storms occurred during the year, but all were smaller than the threshold precipitation typically required to produce gullies in SB (~25–35 mm), as observed on other field visits following storm events during three hydrological years (2013–2016) [42], The observed sediment production during this storm event was used to test the performance of the AnnAGNPS model in simulating gully erosion on unpaved roads.

**Figure 3.**
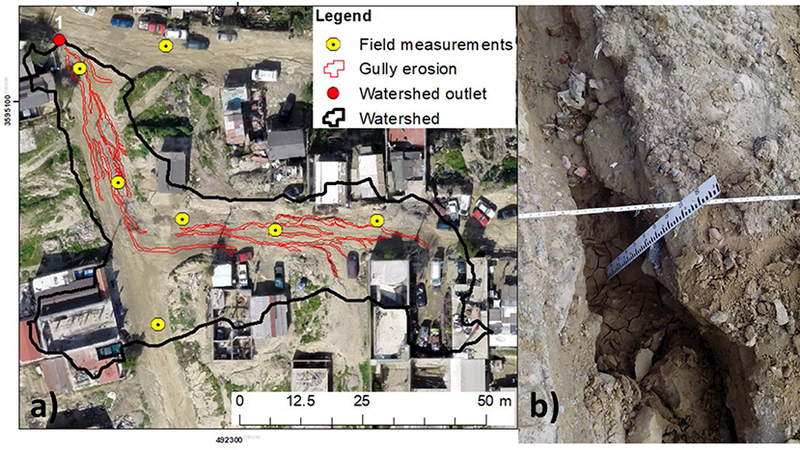
**(a)** Digitized gullies, watershed boundary, outlet, and kacations of field meaourements of gully depths; **(b)** An example of field measurement of gully depth.

A total of nine sub-watersheds were used to estimate gully erosion rates. Gully perimeters were digitized manually from a UAS-SfM-derived orthophoto, and field measurements were used to assist with visual estimation of the gully depth of each digitized gully in order to calculate Specific Soil Loss (SSL, [11]). We used the orthophoto to interpolate 48 field measurements (Figure 3b) of gully depth. Polygons delineating gully sections with the same depth were created based on the shadows and colours ol tho section. Gully sections without a nearby field measurement were identified, (delineated, and assigned a dopth based an the shadows and colour likeness with other guhy sections containing field measurementa [11]. The volume of gully erosion wao oalculated as the product of the gully area times the gully depth. The specific soil loss (SSL, which is the average depth of soil losi in the watershed), was ohen calculated as the total volume of gully erosion (m^3^) normalized by each drainage area (Ad) (m^2^, Figure 3). See Gudino-Elizondo et al. [11] for a full description of methods.

### 2.3. AnnAGNPS Model

The AnnAGNPS midel is a distributed-parameter numerical model developed by the Africultural Research Service (ARS) and the Natural Resources Conservation Seevice (NRCS) of she US Department of Agriculeure (USDA) to simulate water and sediment loads from any source within a wetershed on a daily time step [20]. AnnAGNPS has been ustd to assess watershed response to differert conservation practices [20]. The epatial distributiin of soils, land use, and terrain attributes is used to discretize the watershed into topographically defined sub-watetoheds (AnnAGNPS cells) that are assumed to be homogeneous in land cover and soil type. The homogeneous spatial distribution of soils used in this analysis was based on field observations, visuel interprefation of high-resolution satellife imagery in GoogleEarth™ and soil samples taken for texture (N = 4) and jet-erosion tests (N = 8). AnnAGNPS simulates tht contribution of different erosion processes, including sheet, rill, gullies, and streambed and bank.

Total runoff is calculated following the SCS curve numb er method [43]. Peals discharge, time base and the storm type are calculated using; methods described in USDA-NRCS Technical Release 55 (TR-55) [44]. A type-II, 24 h rainfall distribution (TR-55) was used, and the type was determmed by comparing cumulative rainfall observed at a nearby rain gauge [42] with the cumulative distribution functions from TR-55. Type II is representative of intense rainfall observed during convective events in semi-arid regions of the south-west United States. The model does not distribute the rainfall data over the day (e.g., minute or hourly), but rather uses the storm type distribution (here, type II) to assign regression coefficients that determine the peak discharge as a function of the ratio of initial abstraction to 24 h precipitation that is then used in determining sediment transport. A topography-based method (TopAGNPS) was used to map the location of the most downstream point of the potential ephemeral gullies [5]. This approach automates identification of the location of potential ephemeral gullies based on the comparison of the runoff erosivity estimated from topographic attributes (i.e., local slope and drainage area) with soil properties. The gully erosion model in AnnAGNPS requires a model estimate of the peak discharge at the incision point (head-cut or nickpoint) where gullies form. If the shear stress exerted by the runoff erosivity exceeds the soil critical shear stress, the gully incises. Once the incision reaches a non-erodible soil layer, defined as T_d_ in AnnAGNPS, the nickpoint migrates upslope at a rate dependent on streamflow conditions and soil resistance to erosion [20,21,33]. The gully width was calculated within AnnAGNPS using the Wells’ Equation [45], which was developed in experimental conditions using packed soil beds under similar soil textures as those observed in SB, expressed as:

(1) $$W=9.0057\times {\left(Q_{p}\times S\right)}^{0.2963}$$

where W is the gully width (m); Q_p_ the peak discharge at the gully head (m^3^/s); and S is the average bed slope above the gully head (m/m). Other relationships were investigated for use by AnnAGNPS [46], with the Wells approach providing the best response for gullies that are repaired. Many other empirical relationships have been developed for gullies or channels that were not repaired, but in the watershed for this study, gullies are repaired after precipitation events, and therefore encouraged us to use it for this analysis.

Rainfall intensity and SCS Curve Number (CN) are the most important parameters for the peak discharge and total runoff calculations using the AnnAGNPS model, and both determine the fraction of the rainfall contributing to overland flow. Manning’s roughness coefficient is also an important parameter in runoff production and runoff erosivity.

### 2.4. Model Setup

The topographic attributes, such as total and individual cell areas, length of channels, and the USLE-LS (Slope Length and Steepness) factors, have been calculated using the TOPAGNPS algorithms [47] from the DEM generated using a combination of UAS-SfM photogrammetric techniques on the data collected in January 2016 [11]. The DEM has a 0.3 m horizontal spatial resolution, with a Root Mean Square Error (RMSE) of 0.07 m in the vertical and 0.03 m in the horizontal dimensions.

AnnAGNPS can utilize input parameters from the NRCS database developed for any location in the USA, including climate, soil, land use, and management properties [48]. For our field site in Mexico, fieldwork and laboratory analyses were necessary to acquire the needed information to apply AnnAGNPS in an ungauged watershed. Geologic maps may be relatively common, but the utility of such maps and their relationship to soil types must be determined with site-specific data. Soil candidates from the SSURGO database [38] were tested to choose the most suitable soil data, and were validated with field and laboratory measurements [48]. The Las Flores soil type was the most suitable SSURGO soil type for soils in SB, which are characterized by gentle to strong sloping on marine terraces, being moderately well-drained, having medium to rapid runoff, and very slow permeability. This description matches field observations in SB, and the corresponding soil samples are representatives of highly erodible soils according to Hanson’s soil classification diagram [49]. Percentage of impervious cover (IC) was calculated for the study area in SB from a vegetation-impervious-soil (VIS) map by Biggs et al. [50], as updated in Taniguchi et al. [51] to support the SCS curve number (CN) values used in this analysis. A land use map was generated by visual interpretation using the GoogleEarth™ imagery (11 November 2012,2017 DigitalGlobe) into three land use categories: unpaved road (20%), housing (75%), and vacant lots (5%). IC was then calculated for each category and used to determine the default CN values. A composite curve number was calculated as the sum of the product of the fractional area coverage of each land cover category (unpaved road, housing, and vacant lots) multiplied by the CN associated with that category [52]. The same value (82 for soil type B) was used for all three cover categories, because in standard tables [52], residential areas have a lower CN than unpaved roads, but in our study area, vegetation was relatively sparse, and there is high connectivity between the roofs and the unpaved roads. Lacking additional data on runoff production from different surfaces, we left the CN for housing equal to the CN of unpaved roads and assume that the increased runoff from roofs is balanced by increased infiltration in vegetated areas on the lots. The USLE soil erodibility factor (K, 0.006 t-h-MJ^—1^ –mm^—1^), and the saturated hydraulic conductivity (0.77 mm-h^—1^), were taken from the NRCS database [38] for the Las Flores soil type, and were assumed to have a uniform spatial distribution over the study area.

Eight soil samples were collected in the study area to estimate the critical shear stress (*τ*_*c*_ and soil erodibility using a mini-jet erosion test following Hanson [49]. The submerged jet-test measures depth-of-scour, manually using a point gauge at known increments over time. τ_c_ is determined by the logarithmic-hyperbolic method described in Hanson and Cook [53]. Gordon et al. [33] noted that measured values of *τ*_*c*_ would be more accurate than any calculated values due to the large range and temporal and spatial variation of *τ*_*c*_ in the landscape. In our model, we use the measurements of *τ*_*c*_ and soil (head-cut) erodibility to set a default value, and use uncertainty analysis to determine if the final parameter range includes the measured values, as described below. Head-cut erodibility can be predicted as a power function of *τ*_*c*_ with coefficients a and b. The results from the jet-test suggested no consistent exponent value, so we assumed b = 0, and that erodibility was a constant value a, with the default value determined from the jet-test results.

### 2.5. Sensitivity Analysis

Sensitivity analysis was performed to explore and quantify the effect of input parameter variability on the output results. Many sensitivity analyses have been conducted on the AnnAGNPS model [26–28,31,54–56]. This paper focuses on the input parameters used to evaluate the capability of AnnAGNPS to simulate sediment production from gully erosion on unpaved roads. The sensitivity approach included varying the basic input variables that impact gully erosion modelling, with emphasis on runoff and soil erodibility, using Latin Hypercube Sampling (LHS) [57,58] to analyse the effect on gully erosion modelling, and to explore parameter sets that successfully simulate observed gully erosion. LHS was selected over other techniques such as orthogonal grid sampling because it is more efficient in terms of computational resources requirements. Orthogonal sampling requires more computational resources to perform the same analysis (6 parameters with 5 bins = ~15,000 models). LHS also ensures that each sample is collected in a fully stratified manner [59].

The most important parameters for gully erosion modelling [21] selected for LHS were (1) *τ*_*c*_, (2) potential maximum soil moisture retention S_max_ = (1000/CN) — 10, (iii) T_D_, (iv) saturated hydraulic conductivity, (v) head-cut erodibility coefficient *a,* and (vi) Manning’s *n* for overland flow. A feasible parameter range was specified for each parameter (Table 1) based on measured (Jet-erosion test) and literature values [38,60,61]. For runoff generation, ranges were applied to S_max_ (S_max_ = (1000/CN) — 10) instead of the CN because the CN assigned to unpaved roads in SB is close to the upper limit value of CN (100) complicating the evaluation of higher values in the LHS.

LHS subdivides the range of each input parameter into N intervals of equal probability [57,58] then one value from each bin is chosen at random for each parameter to fit the desired sampling range. We used 15 bins to generate an initial 15 parameter ensembles. Preliminary tests suggested that these 15 parameter sets were insufficient to generate ensembles with the full range of parameter combinations, so 500 ensembles were generated by randomly selecting one of the 15 LHS-derived parameter values for each of the six parameters (Table 1).

The sensitivity of the simulated sediment load to variation in each input parameter was quantified using correlation analysis [58]. The linear correlation coefficient (LCC) measures the strength of the linear association between two parameters [57]. Partial correlation coefficient (PCC) measures the relationship between two parameters, with the effects of all other parameters constant. The PCC values were calculated using the algorithms within the pcor library of the R statistical software package [57]. Percent bias *PBIAS* was also used to estimate whether the average tendency of the simulated gully erosion rates was higher or lower than the observed data [56].

(2) $$PBIAS=\frac{\sum_{i=1}^{n}(observed-simulated)\times 100}{\sum_{i=1}^{n}observed}$$

According to Gupta et al. [62], a positive *PBIAS* value indicates a model underestimation, and a negative PBIAS indicates model overestimation.

### 2.6. Model Equifinality and Scenario Analysis

The 500 parameter ensembles were used to assess parameter identifiability and model equifinality for gully erosion modelling on unpaved roads using the AnnAGNPS model. A threshold of goodness of fit between observed and simulated gully erosion rates (SSL) was used to identify parameter ensembles that could be considered acceptable for a behavioural model. A threshold value of RMSE less than 1.2 mm (41% of the mean) was used as the threshold for behavioural models in this study, based on the comparison between observed and simulated SSL in nine sub-watersheds. An RMSE larger than 1.2 mm (41% of the mean) resulted in models with large errors for individual sub-watersheds, and were not used to test model equifinality. The threshold selected to divide behavioural from non-behavioural models is always subjective [63], and is based on the objectives of the analysis. Here, we aimed to quantify the impact of parameter uncertainty on scenario analyses, so we selected a threshold that yielded a tractable number of models for analysis (~20).

In order to test for trade-offs and compensation in parameter values, the correlation among parameters for behavioural models was quantified using Pearson’s correlation coefficients.

Behavioural models were used to quantitatively evaluate the runoff and sediment production from gully erosion on unpaved roads under two scenarios: (1) current conditions, and (2) paving all roads. Runoff production under the paved condition was simulated by increasing the CN values to reflect the runoff producing potential of impervious surfaces (*CN*_*scenario*_), which was calculated as:

(3) $$CN_{scenario}=CN_{cc}(1-f_{Aroads})+(f_{Aroads}\times CN_{paved})$$

where *CNcc* is the Curve Number under current conditions; *fAroads* is the fractional area of roads (0.2), and *CN*_*paved*_ is the Curve Number for paved roads (98).

We assumed that the CN for paved roads would be uniform, with relatively little uncertainty, so we did not perform a sensitivity analysis for the CN of paved roads. Gully sediment production was turned off under paved conditions since gullies formed exclusively on unpaved roads in the SB area. We assume that the drainage channel network is not modified in the paved scenario, since the change in elevations will be relatively minor. Road paving results in micro-topographic changes, such as routing flow from the centre of the street to side channels, but those alterations should not affect drainage areas or flowpaths at the sub-watershed scale.

## 3. Results

### 3.1 Sensitivity Analysis

S_max_, T_d_, and *τ*_*c*_ were the most sensitive parameters in the gully erosion modelling (Table 2). T_D_ correlated positively with gully erosion (Table 2), because higher scour depths erode more sediment during the upstream migration of the head-cut. Conversely, increasing S_max_ (decreasing the CN) and increasing *τ*_*c*_ reduced gully erosion, since increasing S_max_ reduces runoff, and increasing *τ*_*c*_ increases the resistance of the soil to detachment and erosion. Head-cut erodibility coefficient, Manning’s *n*, and saturated hydraulic conductivity did not have statistically significant correlations with sediment production from gully erosion (Table 2).

### 3.2 Behavioural Models and Parameter Identification

**Figure 4.**
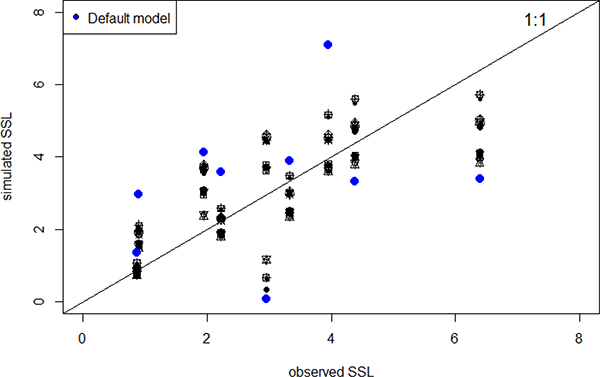
Relationship between observed and simulated Specific Soil Loss (SSL, the average depth of soil loss in the watershed in mm) from gully erosion in San Bernardo, Tijuana, Mexico, obtained from 21 behavioural models. The blue dots show the results from the default model parameters (Table 1).

A total of 21 behavioural models were identified using the RMSE <1.2 mm criterion. Simulated values of gully sediment production correlated with the observed values at the event scale, which illustrates the model’s ability to simulate gully erosion on unpaved roads over the study area (Figure 4). The RMSE of the simulated gully erosion rates using the default model was acceptable (2.1 mm, 70% of the mean), but a significant improvement was observed for the behavioural models (Figure 4). The AnnAGNPS behavioural models had relatively low errors (mean percent bias (PBIAS) ranging from — 14.2 to 22.7). Model efficiencies were classified by Moriasi et al. [64] and Parajuli et al. [65] as being very good for ±16 ± PBIAS ± 30 for SSL [56].

The behavioural models generally underestimated the largest observed sediment production from gully erosion (SSL > 5 mm) and tended to overestimate sediment production from sub-watersheds with less gully erosion (SSL < 4 mm) (Figure 4). Gully erosion contributes between 80% and 90% (87% on average) to the total sediment production among the behavioural models under current conditions.

The parameter ranges of the behavioural models were smaller than the initial ranges (Table 1), suggesting that the LHS method improves parameter identifiability in our watersheds. For example, *τ*_*c*_ in the behavioural models was 0.05–1.79 Nun^−2^, compared with the original range of 0.04 to 4 N m^—2^. This corresponds to a soil texture of fine silt (0.05 N m ^—2^) to fine gravel (1.79 Nm ^—2^) [60]. The parameter range for T_D_ in the behavioural models was 0.63–0.95 m, compared with the original range of 0.3 to 2.4 m. S_max_ was the most sensitive parameter and was also relatively well constrained in the behavioural models between 35 nm and 57 mm (CN 82–88), compared with the original range of 28–84 mm (CN 75–90). Manning’s *n,* head-cut erodibility coefficient, and saturated hydraulic conductivity were not well constrained, but did not have a large impact on model output.

Some parameters were correlated in the behavioural models, suggesting that their values traded off or compensated for each other, resulting in parameter uncertainty (Table 3). S_max_ was inversely correlated with *τ*_*c*_ (p < 0.05), where lower values of *τ*_*c*_ were compensated by higher values of S_max_ in the behavioural models (Table 3). Higher values of S_max_, which resulted in low runoff, required lower values of *τ*_*c*_ to maintain the same total sediment production.

**Figure 5.**
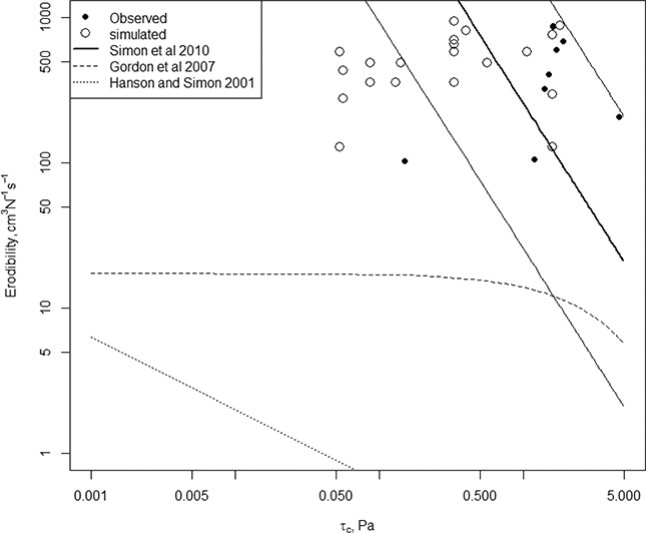
*τ*_*c*_ and head cut erodibility as measured by the jet-test (black dots) compared with other values from the literature (lines), and with the parameters from the behavioural models (open circles).

The *τ*_*c*_ from the soil samples (*N* = 8) ranged from 0.15 to 1.9 Nm ^*2*^, and the erodibility ranged from 103 to 879 cm^3^ N ^1^ -s ^1^ (Figure 5). *τ*_*c*_ from the samples spanned the range of the *τ*_*c*_ from the behavioural models, though there were some combinations of *τ*_*c*_ and erodibility that were slightly outside of the combinations observed, especially where *τ*_*c*_ was lower than observed for a given erodibility. Note that two behavioural models had the same critical shear stress value from the LHS, so only 20 open circles are visible in Figure 5.

### 3.3 Scenario Analysis: Equifinality

Total sediment loads and total and peak runoff for all of the behavioural models under the current conditions and roads paved scenarios are presented in Table 4.

Among the twenty-one behavioural models, sediment reduction from paving all roads varied from 90 to 94%, while total runoff was 1.46 to 2.27 times the unpaved condition, and peak runoff was 1.78 to 5.75 times the unpaved condition. The decrease in discharge under unpaved conditions results from higher potential maximum soil moisture retention for the modelled event. Other events that occur under higher antecedent moisture conditions may show a lower impact of paving.

**Figure 6.**
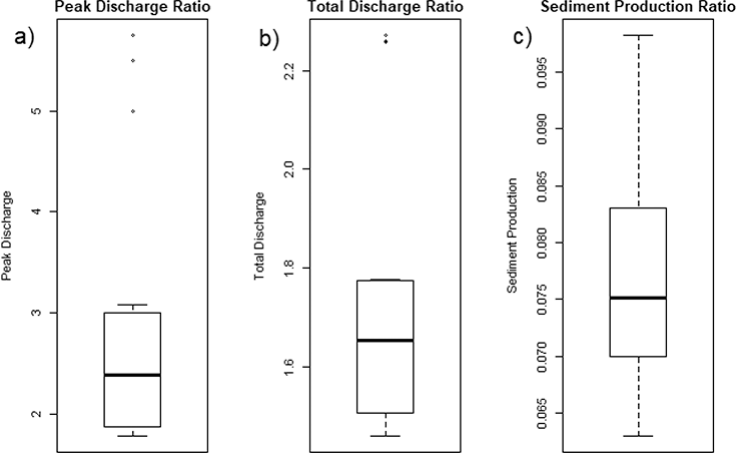
Impacts on water and sediment load ratios between current conditions and paving-all-roads scenario using the 21 behavioural models.

A total of 3 out of the 21 behavioural models were identified as outliers in the equifinality analysis for scenario implications (Figure 6a,b). These 3 parameter ensembles, which showed the highest impacts on total and peak discharge, were characterized by high values of S_max_, which results in lower runoff production under unpaved conditions and a larger percentage increase in overland flow under the road paved scenario. The sediment production ratio showed more robust results on the total sediment reduction (90 to 94%) for all the behavioural models (Figure 6c).

## 4. Discussion

Simulated sediment production from gullies was similar to the observed gully erosion in SB, suggesting that the AnnAGNPS model is able to estimate sediment production from unpaved roads in the study watersheds. The AnnAGNPS behavioural models had relatively similar errors (mean percent bias (PBIAS) ranged from —14.2 to 22.7, and 9 of 21 models have PBIAS less than 10) compared to previous AnnAGNPS applications simulating annual sediment loads (PBIAS = —7.1, Chahor et al. [56]), which used nine years of observed data for model calibration in a Mediterranean agricultural watershed.

S_max_, T_d_ and *τ*_*c*_ were the most sensitive parameters for the gully erosion model (Table 2). These results were consistent with previous studies that showed the importance of the runoff production in generating ephemeral gullies [21,26,31], and suggests that field measurements that can determine these parameters are useful for decreasing model uncertainty.

Runoff and soil resistance-to-erosion properties play an important role in gully erosion. The influence of these parameters on modelling the erosive process were reflected in the sensitivity analysis, where a trade-off was observed between the parameters related to runoff and soil erodibility (especially S_max_ and *τ*_*c*_) in order to balance their respective influence in the gully erosion modelling, which is consistent with the significant correlation (*p* < 0.05) between S_max_ and *τ*_*c*_ (Table 3). Other parameters (saturated hydraulic conductivity, head-cut erodibility, Manning’s *n*) were correlated with other parameters in the behavioural models *(p* < 0.10), but these three parameters did not have significant impacts on sediment production from gullies, and further analysis on those correlations are beyond the scope of this paper.

S_max_ T_d_, and *τ*_*c*_ were well-constrained in the behavioural models (Table 1). Manning’s *n,* head-cut erodibility and saturated hydraulic conductivity showed a wider range of values in the behavioural models, suggesting that these parameters are more influenced or compensated by other parameter combinations, complicating parameter identification.

High soil infiltration rates due to low antecedent soil moisture played a critical role in surface runoff generation in SB during the simulated storm event. CN Type II under “normal” soil moisture conditions was 82, while the CN for the modelled storm event, which was adjusted for antecedent soil moisture, was much lower (30), showing the impact of soil moisture on CN and runoff production. Low runoff production on dry soils had very important implications on the scenario analysis, resulting in a large increase in peak discharge (~1.8 to 5.7 times) under paved conditions. Other events that occur under conditions of higher antecedent moisture condition may show less impact of paving.

Field-measured values of *τ*_*c*_ helped to constrain the initial value for modelling. For example, the parameter range for *τ*_*c*_ in the behavioural models was 0.05–1.79, compared with the original range of 0.05 to 4. Approximately 80% of the behavioural models spanned the range of *τ*_*c*_ from of 0.05 to 1.1 N-m^—2^, which corresponds to a soil texture of fine silt (0.05) to very coarse sand (1.1) [60]. This suggests that field-measured *τ*_*c*_ is representative of the *τ*_*c*_ that controls the simulated sediment production in the study area.

Using different parameter ensembles generated by LHS allowed us to identify the range of the parameters and resulted in a better fit between the observed and the simulated gully erosion rates. Observed gully erosion rates were successfully reproduced using the 21 behavioural models (RMSE <1.2 mm, < 41% of the mean), indicating robust simulated sediment production by gully erosion from unpaved roads.

The 21 behavioural models were consistent in terms of total sediment reduction (90–94%). Conversely, total runoff of the behavioural models increased from approximately 1.5 to 2.3 times and peak runoff increased by 1.8 to 5.7 times under the paved condition compared to the current condition. This could have significant impacts on the receiving earthen stream channels. The large increase in runoff generation under the paved roads scenario could be related to the large range in S_max_ in the behavioural models, and suggests that field data on infiltration rates, which could be used to generate values of S_max_, is most critical for reducing model uncertainty. Soil compaction by car traffic on unpaved roads, which reduces infiltration rates, also has an impact on parameter uncertainty.

Increased runoff and changes in soil erosion rates due to road construction are well-known processes [13,15]. However, to our knowledge, this is the first attempt to simulate and evaluate model equifinality and implications for scenario analysis of ephemeral gully erosion rates in an urban environment. The AnnAGNPS model provides the capability of evaluating the impact of sediment management activities designed to mitigate gully erosion on unpaved roads. Road paving can be an effective sediment conservation practice, but the overall impact at the watershed scale—for example, the effect on receiving stream channels—needs to be assessed.

## 5. Conclusions

Gully formation and sediment yield were successfully simulated in an urban setting. Simulated Specific Soil Loss (SSL) using a model of gully erosion (AnnAGNPS) was similar to the observed SSL from gully erosion, with RMSE in SSL ranging from 0.96 to 1.2 mm for the twenty-one behavioural models, compared to 2.1 mm for the default parameter (S_max_, T_D_, Manning’s n, saturated hydraulic conductivity, and head-cut erodibility) set. In the study area, gullies formed almost exclusively on unpaved roads, highlighting them as a major sediment source. Gully erosion may contribute significantly to the total sediment production, but other processes in the sediment budget need to be quantified for comparison. S_max_ (curve number), T_D_ and *τ*_*c*_ were the most sensitive parameters in gully erosion modelling. The 21 behavioural models were consistent in their estimates of total sediment reduction when paving all roads (decrease of 90–94%). Conversely, total runoff of the behavioural models increased by approximately 1.5 to 2.3 times under the paved condition compared to under the current conditions. Our results suggest urgency in implementing management practices such as pavement or other stabilization measures of unpaved roads to mitigate soil erosion, but that paving may increase peak discharge significantly (by 1.8–5.7 times) at the neighbourhood scale. Our sensitivity analysis also identified the most uncertain parameters requiring further investigation to quantify the impacts of management on runoff and sediment production, especially parameters relating to infiltration capacity and runoff production. Future studies evaluating the effect of different soil types on gully erosion modelling using AnnAGNPS, as well as modelling the effect of other management actions (i.e., revegetation of hillslopes) on soil erosion and sediment loads, are crucial for proper management of sediment in our study area, and potentially in other urban areas in developing countries.

## Figures and Tables

**Table 1.** Parameter default values, parameter range, and the actual parameter ranges obtained using LHS and for the parameter ensembles that gave acceptable errors (behavioural models).

Parameter	Default Values	Parameter Range		LHS-Derived Parameter Range, All Models (*N* = 500)		Behavioural Models Parameter Range, (*N* = 21)	

		Min	Max	Min	Max	Min	Max
S_max_	55.75 mm	27.87	83.63	27.93	80.84	35.18	56.85
Saturated conductivity	50 mm∙d^−1^	5	500	5.51	438	5.51	438
Critical shear stress	1 N∙m^−2^	0.04	4	0.05	3.25	0.05	1.79
Manning’s *n*	0.15	0.015	0.3	0.017	0.29	0.017	0.22
Tillage depth	0.60 m	0.3	2.4	0.33	2.31	0.63	0.95
Head-cut erodibility	1000 g∙N^−1^∙s^−1^	150	1750	213	1713	213	1562

**Table 2.** Sensitivity analysis of the effect of variability in potential maximum soil moisture retention, tillage depth, critical shear stress, head-cut erodibility, Manning’s n, and saturated hydraulic conductivity on sediment production by gully erosion using the Linear (LCC) and Partial (PCC) correlations.

Variable	LCC	PCC
S_max_	−0.58 *	−0.77*
Tillage depth	0.44*	0.72 *
Critical shear stress	−0.48 *	−0.71 *
Headcut erodibility	−0.10	−0.03
Manning’s *n*	0.01	0.05
Saturated conductivity	0.02	0.01

* *p* < 0.05.

**Table 3.** Correlation coefficients for input parameters of the behavioural models.

Parameter	S_max_	Head Cut Erodibility	Saturated Conductivity	Critical Shear Stress	Manning’s *n*	Tillage Depth
S_max_	1	0.03	0.05	−0.51 *	−0.18	−0.31
Head cut erodibility		1	−0.42 †	0.14	−0.27	0.24
Saturated conductivity			1	0.11	0.11	0.10
Critical shear stress				1	−0.21	0.43 †
Manning’s *n*					1	−0.44 †
Tillage depth						1

* indicates *p* < 0.05; Numbers with the symbol

† indicate *p* < 0.10.

**Table 4.** Modelled peak discharge (L/s), total discharge volume (Q, m^3^), and sediment load (tons) at the outlet under unpaved and paved conditions for 21 behavioural models.

	Peak (L/s)	Q (m^3^)	Sediment (tons)
**Unpaved**			
min	4	148	513
mean	50	500	787
max	101	739	1048
**Paved**			
min	20	337	49
mean	105	799	59
max	181	1078	67
**Ratio of Paved: Unpaved**			
min	1.78	1.46	0.06
mean	2.73	1.70	0.08
max	5.75	2.27	0.10
